# Reliability, validity and responsiveness of the EQ-5D in assessing and valuing health status in patients with social phobia

**DOI:** 10.1186/1477-7525-11-215

**Published:** 2013-12-23

**Authors:** Michael Sonntag, Alexander Konnopka, Falk Leichsenring, Simone Salzer, Manfred E Beutel, Stephan Herpertz, Wolfgang Hiller, Jürgen Hoyer, Peter Joraschky, Björn Nolting, Karin Pöhlmann, Ulrich Stangier, Bernhard Strauss, Ulrike Willutzki, Jörg Wiltink, Eric Leibing, Hans-Helmut König

**Affiliations:** 1 Department of Health Economics and Health Services Research, Hamburg Center for Health Economics, University Medical Center Hamburg-Eppendorf, Martinistr. 52, 20246 Hamburg, Germany; 2 Clinic of Psychosomatic Medicine and Psychotherapy, Justus-Liebig-University of Giessen, Friedrichstr. 33, 35392 Giessen, Germany; 3 Clinic of Psychosomatic Medicine and Psychotherapy, University Medicine, Georg-August-University of Göttingen, von-Siebold-Str. 5, 37075 Göttingen, Germany; 4 Clinic and Policlinic for Psychosomatic Medicine and Psychotherapy, University Medical Center, Johannes Gutenberg University Mainz, Langenbeckstraße 1, 55131 Mainz, Germany; 5 Department of Psychosomatic Medicine and Psychotherapy, LWL-University Clinic Bochum, Ruhr University Bochum, Alexandrinenstr 1-3, 44791 Bochum, Germany; 6 Department of Clinical Psychology and Psychotherapy, Johannes Gutenberg University Mainz, Wallstr 3, 55122 Mainz, Germany; 7 Institute of Clinical Psychology and Psychotherapy, Technical University Dresden, Hohe Str. 53, 01187 Dresden, Germany; 8 Clinic of Psychotherapy and Psychosomatic Medicine, Technical University Dresden, Fetscherstr. 74, 01307 Dresden, Germany; 9 Department of Clinical Psychology and Psychotherapy, Goethe University Frankfurt, Varrentrappstr. 40-42, 60486 Frankfurt am Main, Germany; 10 Institute of Psychosocial Medicine and Psychotherapy, Jena University Hospital, Stoystraße 3, 07740 Jena, Germany; 11 Department of Clinical psychology and psychotherapy, Ruhr-University Bochum, Universitätsstr. 150, 44780 Bochum, Germany

**Keywords:** Social phobia, EQ-5D, Reliability, Validity, Responsiveness

## Abstract

**Objective:**

The aim of the study was to analyse the psychometric properties of the EQ-5D in patients with social phobia.

**Methods:**

We used a sample of 445 patients with social phobia with five measurement points over a 30 month period. The discriminative ability of the EQ-5D was analysed by comparing the patients’ responses with the general population and between different disease severity levels. For test-retest reliability we assessed the level of agreement in patients’ responses over time, when there was no change in the Liebowitz Social Anxiety Scale (LSAS). Construct validity was analysed by identifying correlations of the EQ-5D with more specific instruments. For responsiveness we compared the means of EQ VAS/EQ-5D index anchored on improved (deteriorated) health status and computed effect sizes as well as a receiver operating characteristic (ROC) curve.

**Results:**

Compared to the general population, patients with social phobia reported more problems in the dimensions “usual activities”, “pain/discomfort”, and “anxiety/depression” and less problems in “mobility” and “self-care”. The EQ-5D was able to distinguish between different disease severity levels. The test-retest reliability was moderate (intraclass correlation coefficient > 0.6). Correlations between the EQ-5D and other instruments were mostly small except for correlations with Beck Depression Inventory. The EQ-5D index seemed to be more responsive than the EQ VAS, but with only medium effect sizes (0.5 < effect size < 0.8) in the British EQ-5D index and only significant in patients with improved health status. The ROC analysis revealed no significant results.

**Conclusions:**

The EQ-5D was moderately reliable and responsive in patients with improved health status. Construct validity was limited.

**Trial registration:**

Current controlled trials ISRCTN53517394

## Introduction

The EQ-5D is a generic, preference-based index score instrument to measure health related quality of life (HRQOL). The index score is used to compute quality-adjusted life years (QALYs) in cost-utility analysis. Due to scarce resources, economic evaluations are important tools for decision-making on health care resource allocation. Therefore, the instrument used to measure health effects should show good psychometric properties. The EQ-5D has demonstrated its psychometric properties in various diseases and disorders (e.g. inflammatory bowel disease [1], stroke patients [2], schizophrenic, schizotypal, and delusional disorders [3], anxiety disorders [4]). Although the EQ-5D has been used in patients with social phobia [5-8], no validation of the EQ-5D in this patient group has been conducted so far.

Social phobia (SP), also known as social anxiety disorder, is the second most frequent anxiety-mood disorder with a 12-month prevalence rate of 1.9% in Europe [9]. The main symptoms of SP patients are fear of being potentially embarrassed in social or performance situations as well as avoidance of such situations (ICD-10 [10], DSM-IV [11]). The fears may be associated with specific situations like public-speaking (“discrete” or “specific” SP) or with social interactions in general (“generalized” SP). SP has an early onset [12], tends to become chronic [13], and is often accompanied with other psychiatric disorders (such as bipolar disorder, substance abuse disorder, or personality disorder) [14].

The aim of this study was to test the psychometric properties of the EQ-5D in patients with SP. In particular, we analysed the discriminative ability (ability to discriminate between different health states of SP), the test-retest reliability (ability to repeat the similar results when the underlying construct is unchanged), the construct validity (ability to measure adequately the underlying construct), and the responsiveness of the EQ-5D (ability to detect changes given a change in the underlying construct).

## Methods

### Subjects and study design

This study is part of a multicenter randomized controlled trial comparing cognitive behavioural therapy and psychodynamic short therapy for SP (ISRCTN53517394). The trial is part of the Social Phobia Psychotherapy Research Network (SOPHO-NET) [15]. Design and results of the trial have been reported elsewhere [16].

Patients were recruited in five outpatient university clinics across Germany (Bochum, Dresden, Göttingen, Jena, and Mainz), from April 2007 until April 2009. The patient sample can be considered as clinically representative [16]. Inclusion criteria were: (I) diagnosis of SP according to the Structured Clinical Interview for DSM-IV [17] and a Liebowitz Social Anxiety Scale (LSAS) score higher than 30 points [18]; (II) age between 18 and 70; (III) SP being the primary diagnosis based on the severity disorder classification of the Anxiety Disorders Interview Schedule [19]. Exclusion criteria were: (I) psychotic and acute substance-related disorders; cluster A and B personality disorders; prominent risk of self-harm; (II) organic mental disorders; (III) severe medical conditions; (IV) concurrent psychotherapeutic or psychopharmacological treatment [16].

495 patients were randomized to one of the therapy groups (n = 416) or a waiting list group (n = 79). After treatments were completed in the therapy groups, waiting list patients were also randomized to one of the therapy groups and treated as well. Data were collected pre-treatment (T0, n = 495) and post-treatment (T1, n = 364), as well as 6 months (T2, n = 321), 12 months (T3, n = 262), and 24 months (T4, n = 183) after completed treatment (T1). The time interval between T0 and T1 was minimum 6 months but varied due to delays in administrative procedures, vacations, or illness of patients or therapists.

Due to missing data in EQ-5D questionnaires, we used a subsample of n = 445 (t0), n = 329 (t1), n = 288 (t2), n = 244 (t3) and n = 166 (t4) for our analyses.

### Measures

#### ***EQ-5D***

The EQ-5D contains three concepts of expressing HRQOL [20]: (I) The patient-reported “*EQ-5D descriptive system*” has five items, so called “dimensions” (“mobility”, “self-care”, “usual activities”, “pain/discomfort”, “anxiety/depression”). Each of them is recorded with an ordinal three level code (1: “no problems”, 2: “moderate problems”, 3: “severe problems”), resulting in 243 (3^5^) possible health states. These can be expressed as 5-digit codes (e.g. “11233” refers to no problems in “mobility” and “self-care”, moderate problems in “usual activities”, and severe problems in “pain/discomfort” and “anxiety/depression”).

(II) The 5-digit code can be transformed into a utility weight, the so called EQ-5D index. The EQ-5D index is based on a valuation of health states by the general population – indicating the preferences from the general population’s perspective. The *EQ-5D index* ranges to a maximum utility weight of 1 (full health). Death is valued with 0. The worst possible health state (“33333”) is -0.21 for the German EQ-5D index [21] and -0.59 for the British EQ-5D index [22], indicating health states valued worse than death. Both EQ-5D index scores were computed by regression analysis leading to a different valuation of the same health state. In our study we labelled the German EQ-5D index score “EQ-5D index-G” and the British EQ-5D index score “EQ-5D index-UK”. Although we analysed a German patient sample, we used both EQ-5D indexes (being aware of the limited comparability between both populations). The EQ-5D index-G was based on a small population sample (n_German_ = 334 vs. n_UK_ = 2997) and must thus be considered less precise for statistical reasons. The German EQ-5D index scores for all 243 EQ-5D health states were estimated from a sample of 36 health states using a regression model. In contrast to the British EQ-5D index, the German EQ-5D index is insensitive to a change from level 1 (“no problems”) to level 2 (“moderate problems”) in the dimension “anxiety/depression” due to omitted regression coefficients. Therefore, the EQ-5D index-G scores must be considered preliminary.

(III) Patients are asked to rate their current health state on a visual analogue scale (*EQ VAS*) ranging from 0 (worst imaginable health state) to 100 (best imaginable health state). The EQ VAS represents the value of HRQOL from patients’ perspective.

#### ***Liebowitz social anxiety scale (LSAS)***

The LSAS is a 24-item clinician-administered SP screening instrument, measuring anxiety and avoidance [23]. Both subscales (“LSAS avoidance score” and “LSAS anxiety score”) range from 0 to 72, leading to a total range of 0 to 144 (“LSAS total score”). LSAS total scores below 30 indicate remission of SP, scores between 30 and 59 indicate specific SP, and scores above 60 indicate generalised SP [18].

#### ***Social phobia and anxiety inventory (SPAI)***

The SPAI is a self-reported SP screening instrument, measuring the disease severity level of SP [24]. The German version of SPAI contains 22 items [25]. Each item ranges from “never” (coded as 0) to “always” (coded as 6), leading to a 7 point Likert scale. The SPAI score as the mean of all 22 items ranges from 0 to 6 with an increasing severity level of SP.

#### ***Beck Depression Inventory (BDI)***

The BDI is a screening instrument for depression [26]. Patients are asked to rate their feelings throughout the last week and today on 21 items. The items range from 0 to 3 with an increasing disease severity level and are added up to a total score ranging from 0 to 63.

### Analysis

For statistical analysis, we collapsed “moderate problems” and “severe problems” of the EQ-5D descriptive system into one category “problems” (except for analysing discriminative ability related to the general population), because the number of patients indicating “severe problems” was small.

*Discriminative ability* reflects the ability of an instrument to discriminate between different health states [27]. We assumed that the EQ-5D discriminates between patients with SP and the general population and between different levels of disease severity in patients with SP. For the comparison with the general population, we used EQ-5D data from a representative survey (n = 3552) in Germany [28] adjusted for age and gender due to the young age in the patient sample. To distinguish between disease severity levels, we grouped patients into quartiles of the LSAS total score and its both subscales, and alternatively, into patients with specific SP (30 to 59 LSAS total score) and generalised SP (≥ 60 LSAS total score). We tested for significance by using χ^2^-test and Fisher’s exact test (EQ-5D descriptive system) and Mann-Whitney-U-test (EQ-5D index and EQ VAS).

*Test-retest reliability* reflects the ability of an instrument to produce similar results if the underlying construct has not changed [29,30]. The LSAS total score was used as clinical anchor. We assumed that the score of both EQ-5D indexes and the EQ VAS stay constant if the change in LSAS total score stays within range of 0 ± 0.5 standard deviations (baseline) which has been recommended by [31,32], corresponding to 11 LSAS total score points. Additionally, we split the “no change” group into patients with and without social phobia (< 30 LSAS total score points at both time points).

For the EQ-5D index scores and the EQ VAS score, we analysed test-retest reliability using the intraclass correlation coefficient (ICC) with a two way mixed model. We considered an ICC ≥ 0.7 as large [30].

*Construct validity* reflects how appropriately the instrument refers to the underlying construct [30]. We assumed that there is an association between the EQ-5D and instruments of psychopathology used as the underlying construct (LSAS total score, LSAS avoidance score, LSAS anxiety score, SPAI score, SPAI No. 22 score, and BDI score). Since both EQ-5D index scores and the EQ VAS score did not follow a normal distribution, we computed the non-parametric Spearman rank correlation coefficient (r_s_) for both EQ-5D index scores and the EQ VAS score. We defined a correlation as small for 0.1 ≤ |r_s_| < 0.3, moderate for 0.3 ≤ |r_s_| < 0.5, and large for |r_s_| ≥ 0.5 [33].

*Responsiveness* reflects the ability of an instrument to change, given a change in the underlying construct [30]. Again, the LSAS total score was used as clinical anchor. We assumed that both EQ-5D indexes and the EQ VAS score change over time if the LSAS total score has changed. We defined a relevant change as more than ± 0.5 standard deviations (baseline) which has been recommended by [31,32], corresponding to 11 LSAS total score points. The responsiveness can be assessed in many different ways [34-38]. In our analysis we used the paired t-test statistics and computed the effect size (ES) to assess the association of change in both EQ-5D indexes and the EQ VAS with the change in the LSAS total score. According to Cohen, we defined scores of ES as trivial from ≥ |0.1| to < |0.2|, as small from ≥ |0.2| to < |0.5|, as medium from ≥ |0.5| to <|0.8|, and as large ≥ |0.8| [33]. Alternatively, we calculated the area under curve (AUC) of the receiver operating characteristic (ROC) curve. An AUC of 0.5 indicates that the instrument randomly detects the true change of patients’ health status. The closer the AUC equals 1.0 the more the instrument is able to detect the true change of patients’ health status [39,40].

As we tested several hypotheses, a Bonferroni correction for the level of significance was computed. Six different instruments were used for analysing construct validity, leading to a corrected level of significance of α = 0.05/6 = 0.0083. Ten different chronological comparison-pairs were used for analysing reliability and responsiveness. Thus, the level of significance was defined as α = 0.05/10 = 0.005.

Statistical analyses were conducted using Statistical Package for the Social Sciences (version 18, SPSS Inc., Chicago, IL, USA).

## Results

### Patient characteristics

Patient characteristics at baseline are shown in Table 1. Mean age was 35.1 years (SD: 12.1). Most of the participants were female (55.5%), unmarried (67.6%), living with their spouse/partner (41.1%), and had finished the secondary school (68.5%).

**Table 1:** Patient characteristics at baseline (N = 445)

**Characteristics**	
Gender: n (%)	
Male	198 (*44.5*)
Female	247 (*55.5*)
Family status: n (%)	
Unmarried	301 (*67.6*)
Married	104 (*23.4*)
Separated/divorced	38 (*8.5*)
Widowed	2 (*0.5*)
Living situation: n (%)	
Alone	148 (*33.3*)
With spouse/partner	183 (*41.1*)
With relatives	72 (*16.2*)
Other forms	42 (*9.4*)
Education: n (%)	
Low	36 (*8.1*)
Middle	102 (*22.9*)
High	305 (*68.5*)
Other	2 (*0.5*)
Age: mean (SD)	35.1 (12.1)

### EQ-5D scores

In the EQ-5D descriptive system, 81.8% of patients reported problems in at least one dimension. Specifically, 75.4% of patients reported at least moderate problems in the dimension “anxiety/depression”, followed by “pain/discomfort” (35.1%), “usual activities” (27.2%), and “mobility” (5.4%). In contrast, no patient reported problems in the dimension “self-care”. The health state 11112 was the most frequently reported health state (30.3%), indicating moderate problems in the dimension “anxiety/depression”, followed by 11111 (18.2%), and 11122 (16.0%). While the mean EQ-5D index-UK was 0.78 (SD: 0.18), the mean EQ-5D index-G was 0.92 (SD: 0.13). The mean EQ VAS score was 75.21 (SD: 16.8) (Table 2).

**Table 2:** Descriptive statistics of the EQ VAS score, the EQ-5D index-G, EQ-5D index-UK, and the comparators at baseline

**Measures**	**Possible range of score (worst-best)**	**N**^**a**^	**Score**	
			**Mean (SD)**	**Median (range)**
EQ VAS score	0 - 100	438	75.2 (16.8)	80 (25 - 100)
EQ-5D index-G	-0.21 - 1.000	443	0.920 (0.135)	0.999 (0.361 - 1.000)
EQ-5D index-UK	-0.59 - 1.000	443	0.785 (0.178)	0.814 (0.186 - 1.000)
LSAS total score	144 - 0	445	72.2 (22.0)	70.0 (10 - 127)
LSAS anxiety score	72 - 0	445	39.3 (10.9)	38.0 (10 - 67)
LSAS avoidance score	72 - 0	445	32.8 (12.1)	32.0 (0 - 65)
SPAI score	6 - 0	437	4.1 (0.9)	4.0 (1 - 6)
SPAI No. 22 score	6 - 0	421	3.2 (1.0)	3.2 (0 - 6)
BDI score	63 - 0	434	14.2 (9.1)	13.0 (0 - 42)

^a^Number of observations varied due to missing values; BDI: Beck Depression Inventory; LSAS: Liebowitz Social Anxiety Scale; SPAI: Social Phobia and Anxiety Inventory.

### Scores of instruments of psychopathology

Mean LSAS total score was 72.2 (SD: 22.0), indicating marked SP (in detail see Table 2). Mean LSAS avoidance score was 32.8 (SD: 12.1) and LSAS anxiety score was 39.3 (SD: 10.9). The SPAI score displayed a mean of 4.1 (SD: 0.87), indicating moderate SP. Mean of SPAI No. 22 score was 3.2 (SD: 1.04), indicating moderate physiological reactions in social situations. The mean BDI score was 14.2 (SD: 9.1), indicating mild depression.

### Discriminative ability

**Figure 1:**
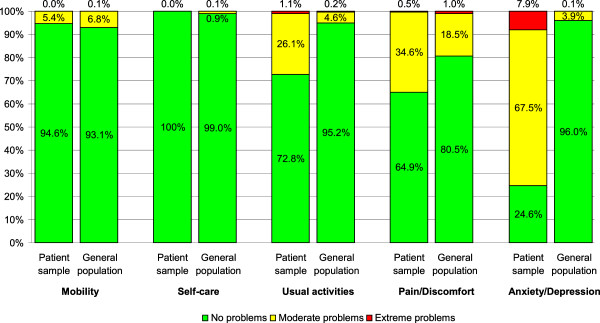
**Comparison of EQ-5D dimensions between patient sample (*n*** **= 445) and general population (*n*** **= 3137).** Data of the general population from [28]. Respondents of the general population were adjusted to age and gender. There were one missing value in “usual activities” (*n* = 444) and two missing values in “anxiety/depression” (*n* = 443) in patients with social phobia.

Compared to the general population, patients with SP reported significantly less problems in the EQ-5D dimensions “mobility” and “self-care” but significantly more problems in the EQ-5D dimensions “usual activities”, “pain/discomfort”, and “anxiety/depression” (*p* < 0.05, see Figure 1).

With increasing disease severity level (according to LSAS-quartiles), the proportion of patients indicating problems continuously increased in the EQ-5D dimensions “usual activities” and “anxiety/depression”, and the EQ-5D index-UK and the EQ VAS score continuously decreased, respectively (Table 3). However, most of these differences were not significant except for the EQ-5D index-UK for which two of three pair-wise comparisons between LSAS quartiles were significant.

**Table 3:** Patients reporting problems in the EQ-5D dimensions (%) and mean EQ VAS score/EQ-5D index scores by disease severity at baseline

		**Mobility (%)**	**Self-care (%)**	**Usual activities (%)**	**Pain/discomfort (%)**	**Anxiety/depression (%)**	**Mean of**		
**LSAS total score**	***n***						**EQ VAS score**	**EQ-5D index-G**	**EQ-5D index-UK**
30 – 55	110	3	0	16	29*	59*	79	0.944*	0.838*
56 – 70	113	6	0	19*	43*	77	78	0.928	0.795
71 – 87	111^a^	4	0	31	29	79	74	0.928*	0.788*
≥88	111^b^	9	0	42	39	86	70	0.879	0.718
Specific SP	137	3	0	16*	34	64*	79*	0.940*	0.828*
Generalised SP	308^c^	7	0	32	36	81	74	0.911	0.766

*p ≤ 0.05 regarding differences to the next group of disease severity; SP: social phobia.

^a^one missing value in “usual activities” and “anxiety/depression”.

^b^one missing value in “anxiety/depression”.

^c^one missing value in “usual activities” and two missing values in “anxiety/depression”.

The analysis of LSAS avoidance score and LSAS anxiety score showed similar patterns for the EQ-5D dimensions. However, we could not find any significant differences in the EQ-5D index scores and the EQ VAS except for both EQ-5D indexes between the third (LSAS anxiety score range from 38 to 47) and fourth quartile (above 48 LSAS anxiety score points) in the LSAS anxiety score (results not displayed).

When comparing patients with specific and generalised SP, patients with generalised SP reported significantly more problems in the EQ-5D dimensions “usual activities” and “anxiety/depression”, and had significantly lower EQ-5D index scores and EQ VAS score compared to patients with specific SP.

### Test-retest reliability

Due to five measurements, we could test the test-retest reliability for 10 different chronologically paired comparisons. For patients with no health status change, the ICC of the EQ VAS score and of the EQ-5D index-G was mostly about 0.6, indicating moderate correlation (Table 4). The ICC of the EQ-5D index-UK was low regarding comparisons including baseline (t_0_), whereas the ICC was mostly large in all other chronological comparisons (ICC > 0.7). The EQ-5D index-UK showed slightly higher ICCs compared to the EQ-5D index-G. Thereby, the group of patients with SP had predominantly higher ICCs in comparison to the group of patients without SP.

**Table 4:** Reliability of the EQ VAS score and EQ-5D index scores anchored by no change of the LSAS total score

				**Intraclass correlation coefficient**								
**Time**	**Number of patients**			**EQ VAS score**			**EQ-5D index-G**			**EQ-5D index-UK**		
	**All**	**SP**	**No SP**	**All**	**SP**	**No SP**	**All**	**SP**	**No SP**	**All**	**SP**	**No SP**
t0 - t1 ( 6 m)	67	67		0.60**	-	-	0.33			0.45		
t0 - t2 (12 m)	44	44		0.53**	-	-	0.56**			0.63**		
t0 - t3 (18 m)	30	30		0.71**	-	-	0.59			0.51		
t0 - t4 (30 m)	21	21		0.65	-	-	0.17			0.34		
t1 - t2 ( 6 m)	181	106	75	0.66**	0.68*	0.58*	0.61**	0.74*	0.17	0.74**	0.78*	0.52*
t1 - t3 (12 m)	145	79	66	0.66**	0.68*	0.52*	0.47**	0.56*	0.06	0.65**	0.65*	0.38
t1 - t4 (24 m)	92	49	43	0.60**	0.65*	0.32	0.74**	0.77*	0.32	0.73**	0.73*	0.46
t2 - t3 ( 6 m)	169	94	75	0.83**	0.81*	0.82*	0.73**	0.72*	0.58*	0.79**	0.78*	0.69*
t2 - t4 (18 m)	104	54	50	0.60**	0.58*	0.59*	0.51**	0.50*	0.49*	0.62**	0.62*	0.55*
t3 - t4 (12 m)	111	60	51	0.72**	0.68*	0.75*	0.67**	0.65*	0.61*	0.78**	0.78*	0.60*

LSAS: Liebowitz Social Anxiety Scale; m: months; SP: Social Phobia. ** p ≤ 0.005; * p ≤ 0.008. No change of the LSAS total score was defined within the range of 0 ± 0.5 SD. Patients without social phobia had a LSAS total score <30 in both time points. In t0 there were no patients < 30 LSAS total score points.

### Construct validity

Both EQ-5D indexes and the EQ VAS score were significantly correlated with the reference instruments, but the correlations were only small (|r_s_| ≤ 0.27; Table 5) aside from a moderate correlation with the BDI score (|r_s_| ≥ 0.44). Additionally, the SPAI score showed a slightly moderate correlation with both EQ-5D index scores (|r_s_| ≥ 0.31).

**Table 5:** Correlation between EQ VAS score, EQ-5D index scores, and scores of other instruments at baseline

	**Correlation coefficients**		
	**EQ VAS score**	**EQ-5D index-G**	**EQ-5D index-UK**
LSAS			
_total score	-0.20*	-0.22*	-0.24*
_anxiety	-0.21*	-0.23*	-0.25*
_avoidance	-0.17*	-0.19*	-0.21*
BDI score	-0.44*	-0.44*	-0.47*
SPAI score	-0.24*	-0.31*	-0.33*
SPAI No. 22 score	-0.18*	-0.16*	-0.17*

BDI: Beck Depression Inventory; LSAS: Liebowitz Social Anxiety Scale; SPAI: Social Phobia and Anxiety Inventory.

Spearman rank correlation coefficient; * p ≤ 0.001; due to missing values the range of observation varied between 410 ≤ *n* ≤ 438.

### Responsiveness

For patients reporting an improvement in their health status on the LSAS total score, both EQ-5D indexes and the EQ VAS score showed significant effect sizes only for comparisons to baseline (t_0_). The effect sizes were mostly small (ES = 0.2 to 0.5; Table 6). The EQ-5D index-UK was the most responsive score (ES > 0.5). For patients with deterioration in their health status on the LSAS total score, we found no significant effect sizes at all (results not displayed).

**Table 6:** Responsiveness of EQ VAS score and EQ-5D index scores anchored by LSAS total score change

		***Improvement of health status***								
		**|Mean difference| (SD**_**mean difference**_**)**			**Effect size**					
**Time interval**	***n***	**EQ VAS score**	**EQ-5D index-G**	**EQ-5D index-UK**	**EQ VAS score**		**EQ-5D index-G**		**EQ-5D index-UK**	
t0-t1 ( 6 m)	235			.073 (.183)					0.44	*
t0-t2 (12 m)	214	3.79 (16.7)	.026 (.132)	.083 (.173)	0.24	*	0.23	*	0.50	*
t0-t3 (18 m)	189	4.51 (16.8)	.038 (.123)	.107 (.175)	0.26	*	0.30	*	0.64	*
t0-t4 (30 m)	129			.091 (.198)					0.53	*

LSAS: Liebowitz Social Anxiety Scale; m: months. * *p* ≤ 0.005. A change of the LSAS total score was defined by more than 0 ± 0.5 SD (baseline). For clarity, we displayed only significant results.

In the ROC analysis, the area under curve was predominantly between 0.5 and 0.6, irrespective of the direction of change of patients’ health status (results not displayed). Furthermore, the area under curve in all time comparisons was not significantly different from the area under the diagonal.

## Discussion

While the psychometric properties of the EQ-5D were analysed in many other diseases and disorders, this study was the first to test the psychometric properties in patients with SP.

The lack of precision of the German EQ-5D index in the dimension anxiety/depression, resulting from the small population sample used to derive the German EQ-5D index, strongly hampers its application in mentally ill patients. Therefore, we also used the British EQ-5D index although British health state preferences may be different from German preferences and may possibly bias our results.

### Discriminative ability

The EQ-5D showed good discriminative ability between the general population and patients with SP. With respect to the EQ-5D dimensions “usual activity”, “pain/discomfort”, and “anxiety/depression”, patients with SP reported significantly more problems than the gender and age adjusted general population which can be attributed to the characteristics of SP such as the fear of social interactions. With respect to the EQ-5D dimensions “mobility” and “self-care”, patients with SP reported significantly less problems than the gender and age adjusted general population. Therefore, one may argue that these EQ-5D dimensions may not have a substantial effect on HRQOL in patients with SP. Problems in mobility may not refer to SP but rather to other co-morbidities.

As there are no validated cut-offs for the LSAS total score available, we used the following two definitions of cut-offs for the LSAS: firstly, we used quartiles, reflecting the severity of SP, secondly we distinguished between specific and generalized SP, reflecting two diagnostic categories.

Using quartiles, the EQ-5D indexes were only able to discriminate between the first and second quartile and between the third and fourth quartile of the LSAS total score scale, whereas the EQ VAS score was not able to significantly discriminate at all. The limited discriminative ability between the second and third quartile may be based on the fact that in second quartile there were patients with severe specific SP and patients with less severe generalised SP.

The EQ-5D was able to discriminate between patients with specific and generalized SP. These findings indicate that the EQ-5D can differentiate to some extent between patients with different disease severity levels of SP depending on the definition of cut-offs of the disease specific instrument.

The results indicated ceiling effects in both EQ-5D index scores and the EQ-VAS score. The ceiling effects result to some extent from the young study sample in which most of the patients had no problems in self-care and mobility. SP may be in general more related to the dimensions “anxiety/depression” and “usual activities” of the EQ-5D. However, the latter dimensions may not distinguish well between patients with mild SP. Thus, some patients tend to report no problems. Both reasons lead to an upward shift of the EQ-5D index scores.

Taken together, we conclude that there is limited evidence that the EQ-5D has discriminative ability in patients with SP.

### Test-retest reliability

We found moderate to large ICCs of the EQ VAS score and EQ-5D index scores in most of the pairwise comparisons. Thereby, the EQ-5D index-UK showed slightly higher ICCs than both other scores. When comparing patients with and without SP, we found that patients with SP had higher ICCs compared to patients without SP. Thus, the EQ-5D seems to be reliable, in particular in patients with SP.

### Construct validity

We identified only small negative Spearman rank correlation coefficients related to the EQ VAS score and the EQ-5D index scores, except for the BDI score and partly for the SPAI score, in which the correlation was moderate. One may argue that both the LSAS total score (and its sub-scores) and SPAI No. 22 score measure different aspects of HRQOL than the EQ-5D. The BDI score and the SPAI score cover some aspects of HRQOL measured by the EQ-5D, namely, the “anxiety/depression” dimension of the EQ-5D. Due to this discrepancy, we may explain this low construct validity of the EQ-5D.

### Responsiveness

We found significant small to medium effect sizes of the EQ VAS score and the EQ-5D index scores in patients with an improved LSAS total score referring to the baseline. In other chronological comparisons we found no significant effect sizes. We assume that treatment effects mainly led to these different effect sizes. Due to therapy, patients improved their health status, leading to a decrease in the LSAS total score. These improvements in health status may be largest compared to baseline and may flatten over time. This indicates that the EQ VAS score and the EQ-5D index scores respond to improvements of health in patients with SP if the improvement is substantial. The EQ-5D may have problems to detect patients with deterioration in their health status, but in our study the sample of patients reporting worse health states was too small to draw any conclusions.

In the ROC analysis, we did not find any significant results. It is possible that the application of ROC curves may be limited in this study due to the small sample size of patients reporting a relevant change.

### Psychometric properties of the EQ-5D in anxiety disorders

We found two articles assessing the psychometric properties of the EQ-5D in anxiety disorders. Lamers et al. [8] analysed discriminative ability and responsiveness of the EQ-5D in a sample of 616 patients with mood and/or anxiety disorders whereas König et al. [4] assessed construct validity and responsiveness in a sample of 389 patients with anxiety disorders.

Regarding the discriminative ability, in the study of Lamers et al. (2006) the EQ-5D index-UK was able to discriminate between different severity subgroups (anchored by the SCL-90 score) except for patients with very severe mood and/or anxiety disorders. In our study, the discriminative ability of the EQ-5D was good in patients with mild and very severe health states. The observed difference in the discriminative ability of the EQ-5D in both disorders may be due to the different properties of the clinical anchors used in the two studies.

Regarding the construct validity, in the study of König et al. (2010) the EQ-5D index-UK and EQ VAS showed large correlations with the BDI scores (r_s_ = 0.59 and r_s_ = 0.54), indicating good construct validity. In our study, the correlation between the BDI scores and the EQ-5D index-UK and EQ VAS, respectively, was similar (r_s_ = 0.47 and r_s_ = 0.44).

Regarding the responsiveness, in the study of Lamers et al. (2006) the responsiveness of the EQ-5D was small (standardised response mean: 0.47). In the study of König et al. (2008) the EQ-5D index-UK showed large effect sizes (0.99) and medium standardised response mean (0.54) in patients reporting worse health status, whereas ES and SRM of the EQ-5D index-UK and EQ VAS were trivial to small in patients with an improved health status. As both cited studies used different clinical anchors for a relevant change compared to our study, the comparability may be limited. Notwithstanding, the responsiveness of the EQ-5D seemed to be similar in patients with an improved health status.

### Strengths and limitations

Our study was based on a large multicenter patient sample resulting in good statistical power. With five different measurements in time we could assess very well the reliability and the responsiveness of the EQ-5D.

However, our study has several limitations. First of all, we could only test the psychometric properties of the EQ-5D with disease specific instruments. Therefore, there may be some limitations in generalisation of our results. Notwithstanding, we could find overall at least moderate psychometric properties indicating that the EQ-5D may be a valid instrument to measure HRQOL of patients with SP. Another limitation of our study was that we had no validated instrument to assess the psychometric properties in the EQ-5D dimension “pain/discomfort”. Thus, we used only a not pain specific instrument (SPAI No. 22 score), indicating just small correlation coefficients in case of both EQ-5D indexes and the EQ VAS score.

### Implications for further research

We suggest three further research topics: (i) The construct validity of the EQ-5D dimension “pain/discomfort” should be reanalysed with a validated instrument. (ii) The EQ-5D should be compared with other generic HRQOL instruments (e.g. SF-36). (iii) The responsiveness in patients with worsened health states should be analysed.

## Conclusion

The EQ-5D seems to be a moderately valid instrument to measure HRQOL in patients with SP. In detail, our study showed a reasonable discriminative ability and reliability. The responsiveness was good as long as the improvement in patients’ health status was clinically substantial. In cases with patients reporting a worse health status, we could not find reasonable results due to small number of patients. Further studies should validate the EQ-5D with another instrument of HRQOL (e.g. SF-36) and for patients reporting a declined health status. Overall, the EQ-5D may be suitable for application in patients with SP.

## Abbreviations

BDI: Beck depression inventory; ES: Effect size; HRQOL: Health related quality of life; ICC: Intraclass correlation coefficient; LSAS: Liebowitz Social Anxiety Scale; QALYs: Quality adjusted life years; SOPHO-NET: Social phobia psychotherapy research network; SP: Social phobia; SPAI: Social phobia and anxiety inventory; SRM: Standardised response mean.

## Competing interest

The authors declare that they have no competing interests.

## Authors’ contribution

MS has made substantial contributions to conception, design, analysis, and interpretation of the data and wrote the manuscript. AK and HHK have made substantial contributions to design, analysis, and interpretation of the data and have been involved in drafting the manuscript. FL, EL, SSa, MB, SH, WH, JH, PJ, BN, KP, US, BS, UW, and JW have made substantial contributions to design and acquisition of data and revised the manuscript critically for important intellectual content. All authors read and approved the final manuscript.
